# Driving accidents and job stress: a systematic review and meta-analysis 

**Published:** 2019-08

**Authors:** Solmaz Azimzadeh

**Affiliations:** ^*a*^Iranian Center of Excellence in Health Management and Department of Health Service Management, School of Management and Medical Informatics, Tabriz University of Medical Sciences, Tabriz, Iran.

## Abstract

**Background:**

Job stress is the interaction between working conditions and individual characteristics. The ‎results of a number of studies in recent years have shown that occupational job stress is an ‎effective factor in the occurrence of traffic driving accidents. Therefore, this study aimed to ‎investigate the effect of job stress on driving accidents.‎

**Methods:**

The present study is a systematic review and meta-analysis that had undertaken in 2019. ‎Keywords were identified using MesH and related articles (for example; job stress,‎‏ ‏occupational ‎stress, work-related stress, Professional Stress, traffic,‎‏ ‏accident, crash, injury). The search had ‎done on valid databases such as PubMed, Scopus, Web of Science, ProQuest, Embace. First the ‎repetitive cases articles were eliminated. Then they were screened by title, abstract, and full ‎text. Then a quality assessment was done for the full texts. The extracted data from the articles ‎entered into Software CMA:2 and the correlation between the two variables of occupational job ‎stress and driving accidents was investigated. The results were displayed illustrated by using the ‎Forest plot and Funnel Plot.‎

**Results:**

7 papers were finally entered into the meta-analysis, though 536 papers. Regarding to the ‎heterogeneity of the results, the random model was used. The correlation coefficient between of ‎occupational job stress and driving accidents was 0.194 (CI) (0.212-0.132), which was ‎statistically significant.‎

**Conclusions:**

Findings showed that job stress is an effective factor in driving accidents. Therefore, ‎recognizing this problem and trying to reduce job stress is a significant issue for managers and ‎policy makers.‎

**Keywords:**

Job stress, Occupational stress, Traffic,‎‏ ‏Accident, Crash

